# Impact of COVID-19 on clinical trials and clinical research: A systematic review

**DOI:** 10.3126/nje.v10i3.31622

**Published:** 2020-09-30

**Authors:** Brijesh Sathian, Mohammad Asim, Indrajit Banerjee, Ana Beatriz Pizarro, Bedanta Roy, Edwin R. van Teijlingen, Israel Júnior Borges do Nascimento, Hanadi Khamis Alhamad

**Affiliations:** 1 Geriatrics and long term care Department, Rumailah Hospital, Hamad Medical Corporation, Doha, Qatar; 2 Centre for Midwifery, Maternal and Perinatal Health, Bournemouth University, Bournemouth, England, United Kingdom; 3 Surgery Department, Trauma Surgery, Hamad General Hospital, Doha, Qatar; 4 Department of Pharmacology, Sir Seewoosagur Ramgoolam Medical College, Mauritius; 5 Cochrane Colombia, Pontificia Universidad Javeriana, Bogotá, Colombia; 6 Department of Physiology, Faculty of Medicine, QIUP, Malaysia; 7 University Hospital and School of Medicine, Federal University of Minas Gerais, Belo Horizonte, Minas Gerais, Brazil; 8 Medical College of Wisconsin, Milwaukee, Wisconsin, United States of America

**Keywords:** COVID-19, pandemic, clinical trial, SARS-CoV-2, research capacity, systematic review

## Abstract

**Background:** The World Health Organization has reported more than 31,186,000 confirmed cases of coronavirus disease-19 (COVID-19), including 962,343 deaths, worldwide as on September 21, 2020. The current COVID-19 pandemic is affecting clinical research activities in most parts of the world. The focus on developing a vaccine for SARS-CoV-2 and the treatment of COVID-19 is, in fact, disrupting many upcoming and/or ongoing clinical trials on other diseases around the globe. On March 18, 2020, the United States Food and Drug Administration (FDA) issued an updated guideline for the conduct of clinical trials during the current health emergency situation. The potential challenges, such as social distancing and quarantines, result in study participants’ inaccessibility and trial personnel for in-person scheduled study visits and/or follow-up. Due to the sudden onset and wide-spread impact of COVID-19, its influence on the management of clinical trials and research necessitates urgent attention. Therefore, our systematic review of the literature aims to assess the impact of the COVID-19 pandemic on the conduction of clinical trials and research. The search for the relevant articles for review included the keywords “COVID-19” AND “clinical trial” in PubMed, MEDLINE, Embase, Google scholar and Google electronic databases. Key findings include: delaying subject enrollment and operational gaps in most ongoing clinical trials, which in turn has a negative impact on trial programmes and data integrity. Globally, most sites conducting clinical trials other than COVID-19 are experiencing a delay in timelines and a complete halt of operations in lieu of this pandemic, thus affecting clinical research outcomes.

## Introduction

The World Health Organization (WHO) declared coronavirus disease-19 (COVID-19) a global public health emergency in late January 2020. COVID-19 is a newly identified coronavirus strain that initially infected people in Wuhan, China [1]. Due to the rapid spread of the infection in China, and other Asian countries, the respective governments have implemented a range of public health preventive measures. The West had experienced the spread of the virus before the Asian countries could control its transmission [2]. Globally, as of the 21^st^ of September 2020, there have been 31,186,000 confirmed cases of COVID-19, including 962,343 deaths, reported by the WHO. The various preventive measures adopted by different countries included travel restrictions (domestic or international), social distancing, with the closure of schools, restrictions on mass gatherings, quarantine, adherence to personal hygiene, use of face masks and, the implementation of infection control measures in healthcare settings [3]. In combination with the lockdown of commercial activities and movement, these restrictions helped to control the spread of infection. However, these global preventive strategies pose unprecedented challenges and obstacles for clinical research; consequently, many clinical research organisations do not initiate new trials, facing difficulties in recruiting new subjects and experiencing lower follow-up rates in the ongoing clinical trials.

The monitoring of patients is possibly the most significant ongoing challenge that the performing trials sites are currently facing. On the 18^th^ of March 2020, the US (United States) Food and Drug Administration (FDA) issued new guidelines for clinical trials’ conduction during this critical situation. The significant obstacles encountered are ‘created’ by social distancing and quarantine measures which forbid interaction between the study participants and trial personnel for study visits and scheduled follow-up. The clinical trial’s integrity is questionable due to the spread of COVID-19 infection to trial participants and staff, which influences trial outcomes with increasing likelihood of trial dropout. Finally, there may be limited access to beds, clinical tests, and Personal Protective Equipment (PPE) as these might be diverted to clinical care facilities and staff to deal with COVID-19 patients.

Due to the sudden onset and widespread impact of COVID-19, its influence on managing clinical trials and research remains undetermined [4-9]. Therefore, this systematic review of research the literature aims to assess the global impact of the COVID-19 pandemic on the conduction of clinical trials and research.

## Methodology

The current systematic review process was performed and reported following the guidelines of the Preferred Reporting Items for Systematic Reviews and Meta-Analyses (PRISMA)[10]. The protocol of this systematic review is registered in the Open Science Framework (OSF# osf.io/2b5zk).

### Literature Searches

This study’s eligibility criteria were articles primarily focusing on the thematic interface between “*COVID-1*9” AND “*clinical trial*”, which were fully indexed in PubMed, MEDLINE and Embase. Furthermore, in order to search for additional eligible records, a similar search strategy was adequately deployed in Google Scholar and Google. The keywords used for searches were variants representing the term coronavirus such as “2019-nCoV”; “2020-nCov”; “ 2019-20 coronavirus*”; “ 2019-2020 coronavirus*”; “SARS-CoV-2”, AND “clinical trial” mentioned either in the title or in the abstract. We have included and screened all published articles highlighted the impact of COVID-19 on clinical trials and clinical research as there is a paucity of literature on this topic due to the sudden onset and evolving nature of the COVID-19 pandemic.

### Inclusion/Exclusion Criteria

The included studies were (a) published original research, (b) written in English; (c) completed and published between January 1, 2020, and September 05, 2020; (d) highlighted the impact of COVID-19 on clinical trial; (e) focused on patients from the global listed countries.

The decision to include or exclude a study was based on the availability of information regarding the impact of COVID-19 on the clinical trial. The relevant studies available as full texts for review were further considered in this review; whereas, abstracts without full texts were excluded.

### Data Extraction

The results of the searches of the databases were initially scanned for appropriate titles of the studies. The selected titles were then screened for the abstracts as well as full texts, and those that met the eligibility requirements were considered for final selection. All the literature evaluation (including abstracts and full-text articles or reports) was independently conducted by four researchers (BS, MA, IB, and BR). The extracted data mainly included the study’s origin, authors, sample size, study population, design, settings/location, period and outcome.

### Methodology Quality Assessment

All systematic reviews incorporate a process of critique or appraisal of the research evidence. The purpose of this appraisal is to assess the methodological quality of a study and to determine the extent to which a study has addressed the possibility of bias in its design, conduct and analysis. We used the Studies The Joanna Briggs Institute (JBI) Critical Appraisal Checklist for Analytical Cross Sectional studies [11].

## Results

**Figure 1:**
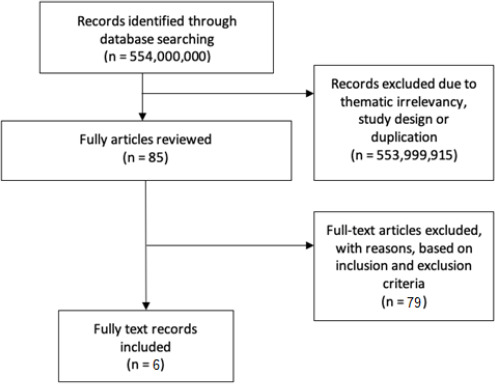
The PRISMA flow diagram for the systematic Review detailing the database searches, the number of abstracts screened and the full texts retrieved.

**Figure 2:**
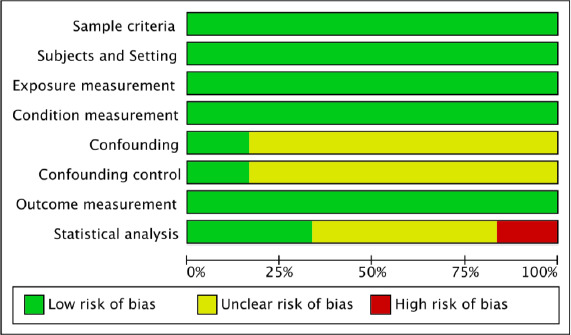
Risk of bias graph:

**Figure 3:**
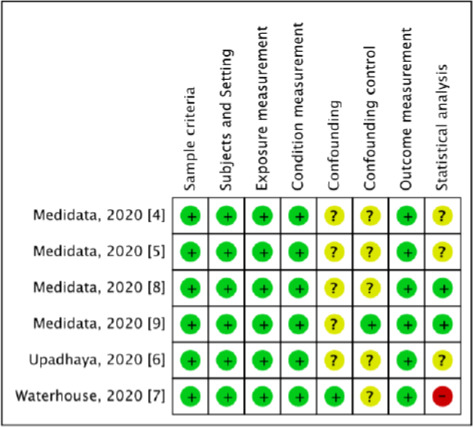
Risk of bias summary

The literature searches generated a total of 554 million articles, of which 553,999,915 were either review articles or duplicate publications that were excluded from the initial screening. Therefore, only the relevant titles and abstracts underwent a detailed assessment, and later on, 81 articles were excluded from the review process that finally resulted in six relevant articles (four research reports and two articles) included in this review. We used the PRISMA diagram to depict the flow of information through the different phases of the systematic review, it maps out the number of records identified, included and excluded (**Figure 1)** [10]. Extracted details for six of the studies are presented in **Table 1** [4-9]. The score consists of eight domains, for figure elaboration and analysis we used RevMan version 5.3 (The Cochrane Collaboration, Copenhagen, Denmark) as shown in **Figure 2 & 3**. Overall quality was high in the studies found; nevertheless, the domains of confounding and strategies to deal with confounding factors were deficient in most of the studies because of presence of some difference between the subjects.

### Characteristics of the studies

On April 23, 2020, the Medidata technology and solutions, a global platform that supports clinical trials, performed an electronic survey of 9,952 staff at various trial investigator sites [5]. This survey had a low response rate of 10.3% as a total of 1,030 participants responded to at least one question of the survey. Most respondents were from the US (North America; 58.3%) followed by Asia (23.8%), Europe (8.1%), South & Central America (7.1%). and the Middle East/Africa (2.7%). The vast majority of respondents were study coordinators (73%), followed by investigators (11.0%), site managers/directors (5.4%), clinical nurses (4.1%) and others (6.3%). The survey results showed that the ability to conduct ongoing clinical trials during the COVID-19 pandemic had affected 69% of respondents, whereas 78% mentioned that this situation affected the initiation of new trials. The primary concerns mentioned by these survey respondents that hamper their ability were subject enrollment (3.7%); recruitment of patients (3.7%); financial losses due to study cancellation (3.4%); and financial implication from delayed endpoints (3.3%), which were computed with a weighted average. Apart from this, other concerns included: accessibility of patients to the trial site (3.1%), health concern of the trial team members (3.1%), and the necessity for COVID-19 screening (3.9%). Overall, respondents were very concerned about subject enrollment and recruitment as more than half mentioned concerns regarding these issues.

Currently, the worldwide impact of COVID-19 on the conduction of clinical trials is being monitored by Medidata. The initial insights and facts about the impact of COVID-19 were reported on March 23^rd^, 2020, which was subsequently updated in April and May 2020. These facts demonstrated a decline in new subject enrollment in many ongoing clinical trials, highlighing the growing impact of this emergency on clinical trials at both regional and international levels with continued regulations for social distancing and guidelines recommending movement restriction outside of the home. In comparison to the pre-COVID-19 baseline (October 31), globally there is around 30% decrease in enrollment of new subjects entering trials by the end of June which was previously higher in April, 2020 (~70% drop). Currently, within different geographic regions, a marked variability has been observed regarding the extent and timing of recovery of patients due to continued fluctuation in the COVID-19 cases, and adoption of regulations over the time [8]. By the end of July 2020, there is an improvement in new subject enrollment in clinical trials per study-site worldwide with around -30% for the month of June in comparison to -6% at baseline (pre-COVID-19). Howevre, the negative effects of COVID-19 on new patients enrollment in clinical trials is expected to continue at different times and varying degree throughout the world which remains understated [9].

The most recent report was released on May 15^th^, 2020 (**Table 2)**, which demonstrated a sharp decline in the average number of new subject enrollment (74%) in clinical trials per study-site year-on-year (YoY) in the initial two weeks of May 2020 as compared to the same period in 2019. This impact on the average number of new subject enrollment for the initial two weeks of May 2020 is comparable to the previous month (April), showing a continued impact of COVID-19 on the new enrollment and clinical trial activities as a whole [5].

**Table 3** highlighted the special considerations regarding different phases of clinical trial conduction in the coronavirus pandemic. In summary, mitigation efforts such as self-isolation and inaccessibility of health care facilities during pandemic interferes with all aspects of clinical trials at various levels. So, the delivery of intervention and collection of outcome data should requires thoughtful considerations. Finally, conduction of clinical trial activities may require suitable protocol amendments to ensure the rights, safety, and wellbeing of the study participants as well as the research staff.

## Discussion

The ongoing COVID-19 pandemic has a potentially negative impact on the management of clinical trials, which may compromise the scientific integrity of data and may raise concerns for patient safety. Moreover, there is an unprecedented operational burden on trials’ conduction as there is limited access to trial activities and investigations of novel therapies or interventions for various diseases, particularly involving vulnerable populations [12,13]. The FDA issued guidelines for conducting clinical trials focusing on this current pandemic which should be followed by research teams currently involved in clinical trial programs [12].

Randomised clinical trials (RCTs) provide the highest quality of evidence for developing newer therapies or interventions to treat challenging diseases, thus improving the quality of life (QoL). Until March 2020, the ClinicalTrials.gov, an international clinical trial registry, documented a total of 262,366 ongoing RCTs, most of which are registered for investigating a drug or biological intervention (n=146,420), followed by trials on behavioural studies (n=85,045) and based on surgical/device interventions (n=61,351) [14].

### Issues in management clinical trials: current perspectives:

For universities and the pharmaceutical industry, useful and timely management of clinical trial operations is crucial due to the substantial financial investment (US$20–50 million per trial). Furthermore, large trial centres usually run multiple trials at any given point of time, enrolling thousands of patients at various sites worldwide. Therefore, this pandemic disrupts operational planning, decision-making, and creates operational gaps that can significantly affect the financial cost and patient safety with interruption of treatment leading to potential harm.

To date, many institutions and organisations have already paused their clinical trials. There are several critical challenges in the current pandemic which include: (a) limited accessibility of clinics mainly for essential or critical visits, (b) there is difficulty in recruiting homebound patients, or others are also reluctant to visit clinics, (c) currently trial operations may expose the staff or patients to the risk of acquiring the infection, (d) continuation of the trial may lead to a high drop-out rate, (e) inability to meet logistical trial obligations by sponsors as well as contractors (i.e., delivery of investigational products, PPE or site monitoring), and (f) deviation from the study timelines may affect data integrity due to delayed assessment and monitoring.

Notably, COVID-19 prevents several institutions from continuing their previously existing trial activities. Disruption of trial programmes has resource, ethical, and care implications. In order to continue the trial operations, it is necessary to follow the regulatory guidelines and regularly review the current infection trend, oversight of lockdown policies and travel restrictions, future prediction of COVID-19 and understanding key risk indicators. Based on these trends, appropriate recommendations and policies should be followed at the trial sites, and risk-based decision making should use evidence from both epidemiologists and health policy-makers. These decisions regarding trials may consider changing study sites, extending programmes, and amendment of planned trial closure, monitoring and screening activities, site access, and, where appropriate, application of machine learning-driven forecasting models. As these changes require time and resources, so it is crucial to have robust risk/issue trackers together with the better symbiosis between operational- and strategic-level decision-making.

Notably, the concept of telemedicine exists for the past two decades. However, its integration into clinical practice has specific administrative and bureaucratic challenges, mainly involving the cost of implementation and reimbursement that is far beyond the conceptualization or technological shortfalls [15]. However, the use of telemedicine approaches have become progressively more popular and acceptable by health authorities, medical doctors and patients after the current pandemic, and positive outcomes from associated intervention have been intensively described and reported worldwide [16,17].

We suggest that adopting telemedicine in the clinical research settings, assessing the global impact of decisions, developing contingency plans, providing additional financial support, greater flexibility and understanding may be useful to help solve this problem. Clinical trials necessitate colossal investment in terms of finance, logistics and other resources (including participants’ time). Similar to the previous pandemics, the impact of the COVID-19 on clinical trials will hopefully be short-lived; meanwhile, to maximize the public health benefits of existing trials, we need creativity and persistence in these unprecedented times.

### Guidelines to be followed on clinical research analysis and conduct during COVID-19 pandemic:

Participants must be self-isolated, and research staff needs to work remotely to avoid direct contact. A framework for detection, recording, assessment, and reporting of all procedural deviations is required, and the appropriate board should be in place to monitor the progress of the clinical trial site(s). All protocol deviations that could lead to severe risk for the patient must be reported. The trial must always be conducted in-line with the Good Clinical Practices (GCP) guidelines.

Monitoring activities should be reassessed to ensure the safety of study participants. Besides, some processes may have to be modified, such as electronic alternatives for signatures that may have to be considered. As administrative personnel may not be able to meet study teams or answer direct calls personally, the use of teleconferencing and voice mails during the pandemic will be useful.

Isolation/quarantine criteria can lead to deviations intended to remove a research participant’s immediate apparent threats. For example, if the participant is unable to attend the treatment appointment, and if the only re-scheduling date available is outside of the study visit period duration, no research protocol or doses should be missed. In this case, a missed dose or procedure for the maintenance of the security and welfare of the subject should not cause any potential harm. So, the health and safety of the participants in the study should be maintained and prioritized. Also, participants must be aware of the risks/changes associated with the study protocol that could affect their well-being. All participants affected by a COVID19-related study disruption should be documented by a unique participant identifier and description of how the individual’s participation was altered. In the case of a visit to the clinical trial site, the researchers may have to assess whether alternate safety assessment measures are feasible if participants are unable to come to research sites as set out in the study protocol.

Alternative methods may include telephonic conversation or the use of telemedicine for virtual visits or alternative sites for care/collecting study data. Alternative locations may need to be considered for imaging and laboratory testing as per study protocol. In the case of alternative monitoring, careful documentation will be required to obtain the reason, how information has been collected, what data has been collected, who has provided the information; how the information sources have been verified and why. No amendments to the study protocol are required.

Researchers are encouraged to consider whether they should hold all the study procedures or part of it during the pandemic. This can refer to all procedures or part of a study. Maybe it is necessary to hold registration, study visits, collect or analysis of data, etc. It is understood that the cessation of the treatment of a patient might not be unsafe or may seriously affect project performance. So, the decision to place a hold on a particular study needs to be made in consultation with the institutional governing body to safeguard the study participants’ and departments’ needs. [18, 19]

Finally, in order to efficiently address the anticipated circumstances by data monitoring committees during the pandemic, special provisions should be placed. For instance, data assessment regarding the benefits and risks of interventions for COVID-19 requires faster communication during health emergencies [20]. This could be possible through virtual meetings with the help of encrypted communication to protect data integrity. Also, most essential measures regarding benefits and risks should be given preference for data capture and reporting to the data monitoring committee for efficient and timely oversight in the COVID-19 setting.

### Expert opinion

Clinical trials are of paramount importance for the advancement and development of novel treatment interventions. The ultimate goal of such trials and studies is to ensure the best quality patient care with the highest and most favourable outcome, whilst decreasing the cost and suffering of (a burden to) the recipient. Such endeavours are vital to the medical community. For the functioning of a robust medical system wherein discoveries, drugs and technology can be applied to the system from the top tier and eventually diffuse out to primary and satellite care centres to ensure the best care for all.

Historically, clinical trials have usually been a laborious, expensive, and difficult task to undertake under normal circumstances; however, trials under the unprecedented COVID-19 conditions have indeed suffered its consequences.

The often complicated design and intricate nature of clinical trials mean the likelihood of derailment is high. A cohort of multiple external factors has to be controlled and maintained to ensure the sanctity of the data as well as the integrity of the trial. All the criteria mentioned above are nullified by a pandemic such as COVID-19.

For the commencement of a new clinical trial or even the maintenance of an existing trial, funding is a priority in order to drive it to completion. COVID-19 severely impeded funding of non-COVID-19 related trials as the acquisition of a cure for the virus took centre stage for international funding bodies. Over and above the funding issue, the need to tightly monitor trial participants and subsequently ensure their safety through such a pandemic proved detrimental to many ongoing studies, or those recently started. The statistics show a sharp decline in the enrolment of new members for trials.

Clinical trials did not only compete for funding under COVID-19 conditions but simultaneously had to cope with lockdown regulations that made accessibility of the study participants virtually impossible. The fact that data from the ongoing studies will be incomplete or will have gaps could lead to either shutting down the trial, a severe delay (and hence additional cost), or the need to re-start the trial. The ramifications of COVID-19 causing such a massive international dearth in the commencement of new trials and the continuation of non-COVID-19 trials will not be evident in the immediate future. However, the impact thereof will become evident as the time lost will add up to years lost in research in the broader spectrum.

COVID-19 and its deleterious effects on trials call for a possible re-structuring of how trials are conducted. A feasible, practical and scientifically sound alternative to conventional trial studies is needed. The alternative will need to ensure scientific integrity but will also need to balance the needs of the parties involved to overcome various difficulties due to external factors. A solution to trials and research as well as time and funding lost during lockdown due to COVID-19 must be addressed, ensuring scientific breakthroughs and the development of new technology is not hindered or delayed. The lives of millions of patients are dependent on the continual clinical breakthroughs and discoveries which are produced by these trials.

## Conclusion

The COVID-19 pandemic has resulted in a series of public health policies that have crippled the healthcare systems of many countries. This situation hugely impacted the study participants, care providers, researchers, trial sponsors, and research organizations concerning clinical trials. This pandemic has a substantial impact on the trial sites as they experience difficulty in the continuation of trial activities which eventually hampers the progress of the trial and delays study timelines. Most sites are struggling due to delayed subject enrollment, shortfalls in monitoring, and risks of compromised data integrity, and this situation has negatively impacted the initiation of future trials as well. Researchers are also concerned regarding the delay or cancellations of trials in this situation, which will have a financial impact on research organizations and human resources. According to one survey, about two-thirds of the respondents have stopped or will soon halt subject enrollment in ongoing clinical trials, one-third halted randomization, and fifty percent of respondents are delaying or planning to delay the studies. Therefore, using dynamic, proactive strategies and a framework for decision-making and risk assessment is needed to overcome these challenges in conducting clinical trials. Adopting new approaches and understanding the key risk indicators will help managers support trial sites with flexibility and ingenuity. For instance, switching patient site visits to new-trial virtualization, and telemedicine to interact with patients will help manage current clinical trials also beneficial for the post-pandemic era.

## Figures and Tables

**Table 1:** Main characteristics of the studies

Author, year	Origin	Study duration	Study design	Sample size	Sampling	Main findings
**Medidata, 2020 [4]**	Multicenter	April 23^rd^, to April 29^th^, 2020	Cross-Sectional	1,030 subjects	Convenience sampling	For 69% of respondents, COVID-19 has affected their ability to conduct ongoing trials, while 78% believe that COVID-19 has impacted the initiation of new trials. The top four concerns based on the weighted average of the answers were: ability to enroll patients (3.73); ability to recruit patients (3.66); financial implications for cancelled studies (3.42); and financial implication from delayed milestones (3.29).
**Medidata, 2020 [5]**	Multicenter	March, April and the first two weeks of May	Cross-Sectional	4,667 studies and 186,807 study-sites	Convenience sampling	A 74% decrease in the average number of new patients entering trials per study-site year-over-year during the first two weeks of May compared to last year. Shows the pandemic continues to have an effect on trial activity and new patients entering trials.
**Upadhaya, 2020 [6]**	Multicenter	March 23^rd^ and April 3^rd^ 2020	Cross-Sectional	36 subjects	Convenience sampling	Patient enrolment in active oncology clinical trials was negatively affected at the time of the survey. Asia [60%], United States [20%] and Europe [14%] were continuing to enrol patients at the usual rate.
**Waterhouse, 2020 [7]**	USA	March 24^th^ to 30^th^, 2020	Cross-Sectional	32 subjects	Convenience sampling	Over half of the respondents (54.8%) observed a decrease in patient’s willingness to come to their site and cited the staff time needed to conduct telehealth visits as a significant challenge. 51.6% noted that limited availability of ancillary services was challenging. Time spent in discussion with sponsors, CROs, and IRBs about modifying trial procedures also presented a challenge for 51.6% participants.
**Medidata, 2020 [8]**	Multicenter	June 15, July 13, 2020	Cross-Sectional	1,030 subjects	Convenience sampling	In comparison to the pre- COVID-19 baseline (October 31), globally there is around 30% decrease in enrollment of new subjects entering trials by the end of June which was previously higher in April, 2020 (~70% drop). Currently, within different geographic regions, a marked variability has been observed regarding the extent and timing of recovery of patients due to continued fluctuation in the COVID-19 cases, and adoption of regulations over the time.
**Medidata, 2020 [9]**	Multicenter	July 13 - August 12, 2020	Cross-Sectional	5,089 studies and 194,506 study sites.	Convenience sampling	By the end of July 2020, there is an improvement in new subject enrollment in clinical trials per study-site worldwide with around -30% for the month of June in comparison to -6% at baseline (pre-COVID-19). The negative effects of COVID-19 on new patients enrollment in clinical trials is expected to continue at different times and varying degree throughout the world which is likely understated.

**Table 2:** Impact of COVID-19 on new subject enrollment in clinical trials

Variables	Variables	YoYD* March 2020 Vs March 2019	YoYD* April 2020 Vs April 2019	YoYD* May 2020 Vs May 2019
**All countries, All Therapeutic areas**		-65%	-79%	-74%
**Asia**	China	-68%	-33%	-49%
	India	-84%	-97%	-95%
	Japan	-44%	-69%	-72%
	South Korea	-61%	-42%	-54%
**Europe**	France	-68%	-81%	-76%
	Germany	-33%	-77%	-81%
	Italy	-53%	-49%	-65%
	Spain	-68%	-82%	-68%
	United States	-66%	-83%	-73%
	United Kingdom	-80%	-95%	-100%
**Therapeutic areas**	Cardiovascular	-69%	-95%	-91%
	CNS**	-68%	-76%	-75%
	Endocrine	-64%	-91%	-89%
	ID/ Anti infectives	-47%	-66%	-52%
	Oncology	-48%	-60%	-58%
	Respiratory	-34%	-86%	-81%

* YoYD = year-on-year difference ;

** **CNS** = central nervous system

**Source:** COVID-19 and Clinical Trials: The Medidata Perspective, Release 5.0 [5]

**Table 3:** Special considerations for conducting clinical trials in the Coronavirus Pandemic

Discovery/ Recruitment	Enrollment/ Participation	Completion/ Follow-up
Coronavirus mitigation efforts (self-isolation) interfere with all aspects of clinical trials at multiple levels.	Efforts and resources should be dedicated to support continuing randomized trials using creative and thoughtful methods and proactive planning.	Effective communication by research staff is likely to help protect against dropout or nonadherence during the pandemic.
Interruption of supply chains and monitoring of clinical trials are additional obstacles.	Research staff should keep participants informed about the effects of the coronavirus pandemic on their trial participation.	Outcomes should be prioritized, and trial operations should be virtualized, if possible.
Ethics committees must apply rigorous standards to authorize research in accordance with the principles of justice, equity and solidarity.	Participants should be informed of necessary changes in protocol and how this may affect the risk associated with study participation.	Alternative methods for measuring primary outcomes should be prepared and protocols modified to facilitate collection of self-reported or medical record data.
Conduction of clinical trials may require modifications to ensure the rights, safety, and wellbeing of participants as well as medical staff.	Delivery of intervention requires thoughtful consideration, emphasizing safety and feasibility under coronavirus restrictions.	Questionnaires previously collected in-person can be converted to telephone administration.
Discontinue randomized trials that do not have an immediate clear benefit to enrolled participants.	Adapting protocols to facilitate continued intervention adherence and outcome measurement.	When feasible and safe, objective outcomes could be collected at home.
